# Case report: A young man with non-rapid eye movement parasomnias in a *KCNT1*-related epilepsy family

**DOI:** 10.3389/fneur.2023.1280348

**Published:** 2023-11-24

**Authors:** Dandan Sheng, Yefan Lv, Xinru Li, Jing Liu, Weiping Liu

**Affiliations:** ^1^Department of Neurology, Xiangya Hospital, Central South University, Changsha, Hunan, China; ^2^National Clinical Medical Research Center for Geriatric Diseases (Xiangya Hospital), Central South University, Changsha, Hunan, China

**Keywords:** sleep-related hypermotor epilepsy, NREM parasomnias, *KCNT1*, nocturnal frontal lobe epilepsy, disorders of arousal

## Abstract

Differentiating between non-rapid eye movement (NREM) parasomnias and sleep-related hypermotor epilepsy (SHE) is challenging, as they exhibit similar episodes during sleep. A relatively high prevalence of NREM parasomnias has been detected in families with SHE. However, the common pathophysiologic mechanism is not completely clear. There have been no previous reports of *KCNT1*-related SHE combined with NREM parasomnias. In this report, we describe a 17 years-old male patient from a *KCNT1* mutation family who exhibited complex abnormal behaviors during sleep, which have been confirmed as epileptic seizures combined with NREM parasomnias through video-electroencephalogram (vEEG) and video-polysomnography (vPSG). The present article provides a reasoning process to evaluate unusual nocturnal behaviors. Furthermore, our analysis suggests a new potential association between NREM parasomnias and *KCNT1* mutations.

## Introduction

Non-rapid eye movement (NREM) parasomnias, also named disorders of arousal (DoA), include confusional arousal (CA), sleepwalking (SW), and sleep terrors (ST), which are classified by the duration, complex behavior type, and degree of forgetting about the onset events (1). They are caused by incomplete separation of wakefulness and NREM sleep, usually arising from slow-wave (N3) NREM sleep. Conditions that stimulate repeated cortical arousal and/or promote sleep inertia lead to NREM parasomnias by impairing normal arousal mechanisms (2). While familial clustering is common in NREM parasomnias, the precise pattern of inheritance remains unclear (1). Differentiating between NREM parasomnias and sleep-related hypermotor epilepsy (SHE) is challenging, as they exhibit similar episodes during sleep.

The *KCNT1* gene is involved in encoding sodium-gated potassium channels (Slack, KNa1.1), and almost all *KCNT1* gene variants increase potassium channel function. Increased sodium-gated potassium current promotes the increase of the discharge rate of the action potential by shortening the duration of the action potential and increasing the amplitude of the post-hyperpolarization potential, and finally increases the excitability of neurons and neural networks (3). *KCNT1* gene variation is closely related to the occurrence of various types of epilepsy, including epilepsy of infancy with migrating focal seizures (EIMFS), autosomal dominant sleep-related hypermotor epilepsy (ADSHE), developmental and epileptic encephalopathies other than EIMFS, as well as other types including temporal lobe epilepsy, epilepsy with tonic-clonic seizures and cognitive regression, and multifocal seizures (4–6).

In this report, we have elucidated the diagnostic process concerning a 17 years-old male patient belonging to a *KCNT1* mutation-related epilepsy family, who also presented with NREM parasomnias. To the best of our knowledge, no prior literature has documented the co-occurrence of *KCNT1*-related disorders and NREM parasomnias.

## Case description and clinical reasoning

A 17 years-old male individual was admitted to our epilepsy ward due to abnormal behaviors during sleep persisting for over 4 years. At the age of 13 years, the patient began exhibiting nocturnal behaviors, such as getting up to open and close doors, with no triggers. These episodes lasted for approximately 1 min, after which the patient would return to bed and fall asleep again. During these episodes, the patient was unresponsive. Typically, these episodes occurred approximately 2 h after falling asleep, happening about once a week initially. However, the frequency of events significantly increased over time, occurring seven times every night. Occasionally, the abnormal movements were limited to the bed area, characterized by sudden sitting up, involuntary hand movements, aimless adjustment of the quilt, confused looking around, and subsequent self-initiated falling asleep that could not be recalled. The patient had not experienced any seizures during wakefulness.

The patient’s younger brother mainly presented with focal to bilateral tonic-clonic seizures during sleep when he was 3 years old. The EEG showed signs of focal onset epilepsy, and oxcarbazepine proved to be effective. Neither the patient nor his younger brother exhibited cognitive impairment, their academic performance was average, and they were able to perform tasks normally. The patient has no other siblings. The patient’s mother experienced sudden awakenings during the night when she was 7 years old, feeling frightened and rushing out of the room. She could partially recall the process, and these episodes typically occurred shortly after falling asleep. They happened every few days and persisted for 2 years. No examination or treatment was sought, and the symptoms resolved on their own. The patient’s father, grandparents, and other relatives have not displayed any abnormal symptoms.

At the age of 16 years, the patient underwent the first 24 h vEEG monitoring in another hospital, which did not reveal any noticeable abnormalities as no events occurred during the monitoring period. Additionally, the high-resolution magnetic resonance imaging scan of the brain did not show any abnormalities. The condition was initially considered as parasomnias, and his symptoms were completely relieved after oral administration of Lorazepam 0.5 g before sleep for more than 10 days. However, the episodes recurred, albeit being limited to movements within the bed area similar to previous occurrences. He did not use any other antiseizure medications besides Lorazepam. The frequency of events gradually increased to almost 6 times per night. He had no other medical history or history of medication use before he began taking lorazepam.

Complex and unusual behaviors in sleep typically occur as a manifestation of SHE and parasomnias. In this patient, the behavior of opening and closing doors like mimicking daily activities tended to be sleepwalking. These episodes initially occurred once a week, aligning more with the frequency pattern characteristic of NREM parasomnias. However, as it evolved to seven times per night, it raised the possibility of seizures. Therefore, we could not rule out the possibility that the patient initially experienced only NREM parasomnias and subsequently developed seizures. It was also hard to tell whether the movements limited to the bed when he sat up were an epileptic episode with complex movements or rising arousal movements (RAMs). RAMs are one of the DoA motor patterns, which are characterized by trunk flexion followed by sitting with feet in or out of the bed (7). Unfortunately, normal brain imaging and interictal EEG could not identify the differences between the two. Lorazepam could not either, because it had a therapeutic effect on both. The mother’s performance was suspected of sleep terrors or epileptic panic events. So we thought the patient had a definite family history of epilepsy and a suspicious family history of DoA from his brother and mother.

**Figure 1:**
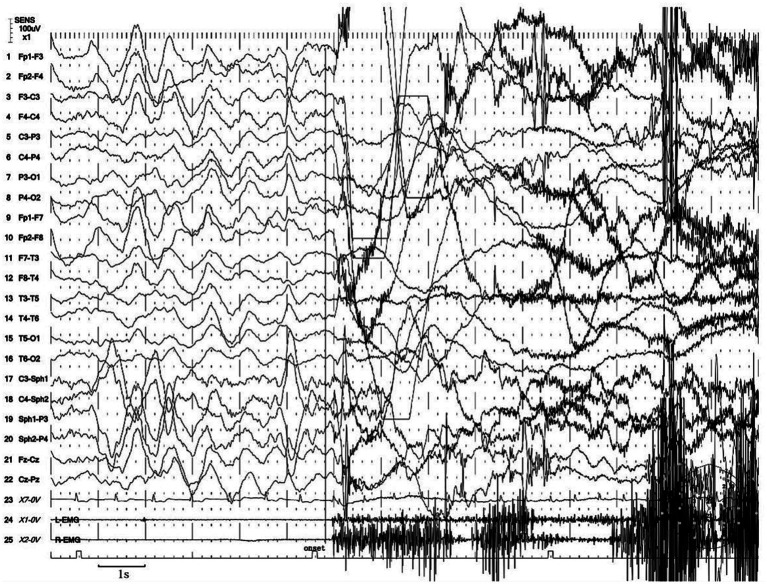
Excerpt of vEEG showing a hyperkinetic seizure. The patient awoke from sleep, sat up abruptly, looked left, exhibited dystonia of the left lower limb, fumbled with hands involuntarily, arranged the quilt aimlessly, and exhibited confusion. The ictal EEG is characterized by a sudden decrease in the amplitude of each area on both sides at the beginning of the event, low amplitude fast waves, synchronous heart rate increase, and a myoelectric burst.

Next, the patient underwent a 24 h vEEG again and a 12 h vPSG monitoring. The vEEG detected four similar episodes during the N3 sleep stage (at 23:48, 00:22, 01:40, and 02:45). These episodes revealed the patient awakening from sleep, abruptly sitting up, looking left, exhibiting dystonia of the left lower limb, involuntarily fumbling with hands, aimlessly arranging the quilt, and exhibiting confusion. Each episode lasted for 80 s (Supplementary Video S1). The EEG during these episodes showed a sudden decrease in the amplitude of each area on both sides at the beginning of the event, low amplitude fast waves, synchronous heart rate increase, and a myoelectric burst (Figure 1).

**Figure 2:**
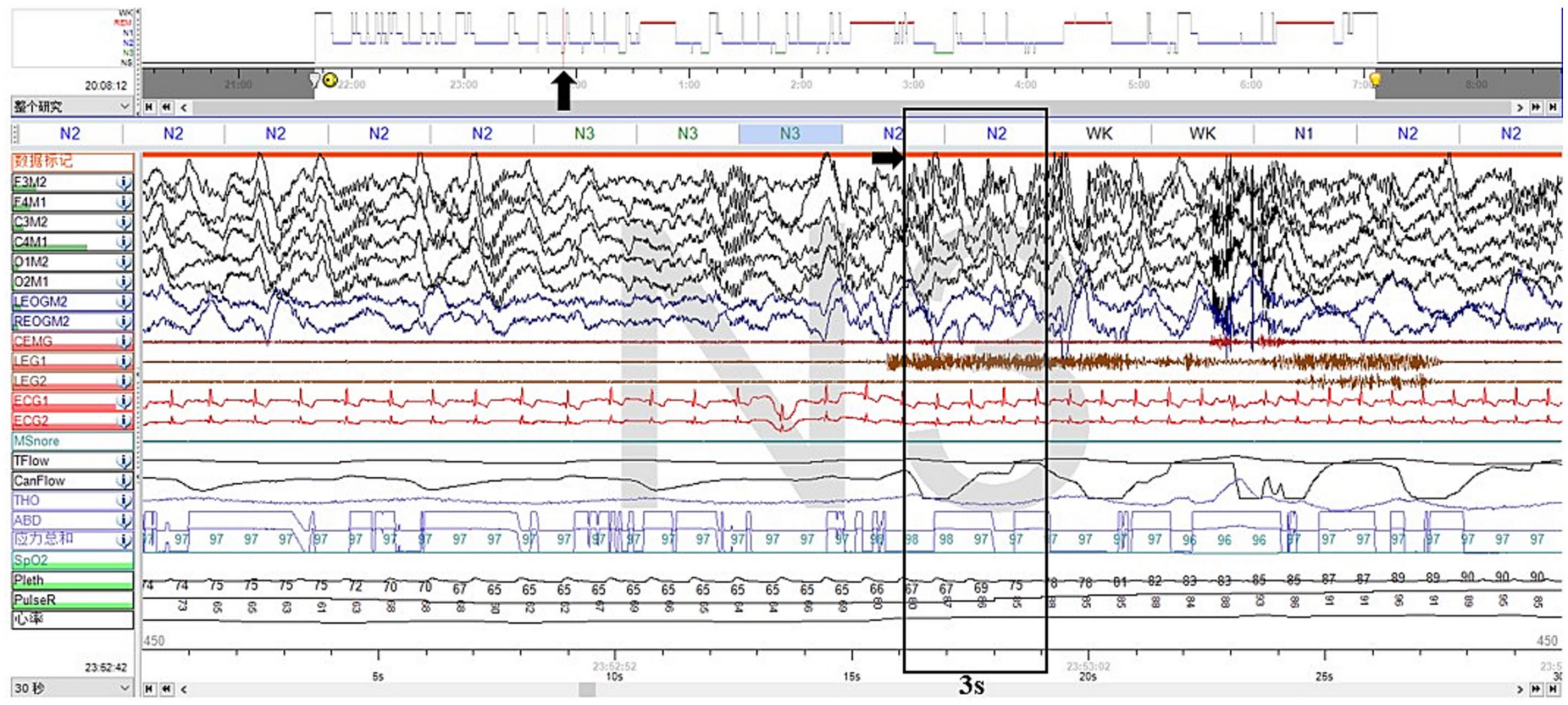
Excerpt of vPSG showing one of the mixed arousal patterns of N3 sleep. The EEG characteristics of the first 3 s of a SWS interruption were mixed delta, theta, alpha, and beta activities.

Furthermore, we noticed two abnormal arousal episodes during N3 sleep in the video, during which the patient slowly raised his head, appeared confused, and looked around, then fell back asleep after approximately 50 s (Supplementary Video S2). The synchronized EEG revealed that slow-wave sleep was interrupted, but did not show any epileptic features. The duration and proportion of N3 sleep appeared reduced, with a SWS fragmentation index of 17.9/h, a mixed/slow awakening pattern of 3.6/h (Figure 2), and high values of cycle-alternating pattern (CAP) rates.

We considered the diagnosis of the event captured in Supplementary Video S1 to be indicative of an epileptic seizure based on the following reasons. First, the episode exhibited a stereotyped and repetitive pattern, with a high frequency of occurrence before treatment, typically averaging four to seven episodes per night. Second, the patient demonstrated lateralization of symptoms, such as looking left and exhibiting dystonia in the left lower limb during the episode. Notably, dystonia is commonly observed in epilepsy, while it is not a feature of parasomnias. In addition, the episode manifested as continuous and progressively involved multiple body regions, resulting in complex movements. Furthermore, most of these events occurred during N3 sleep, even though epileptic seizures originating from N2 sleep are more common. These characteristics collectively supported the diagnosis of epilepsy. Notably, concerning the diagnosis of SHE, the most characteristic seizure presentations include hypermotor movements, characterized by complex limb movements such as kicking, flailing limbs, or body rocking. Asymmetric tonic/dystonic seizures with or without head/eye deviation are also observed. In addition to these, stereotyped arousals including paroxysmal arousal or minor motor events and infrequent seizures involving epileptic nocturnal wandering are also presentations of SHE. Moreover, for many patients with SHE, both the interictal and ictal scalp EEG findings may provide limited information, particularly when seizures originate from deep-seated cortical regions (8).

Supplementary Video S2 more closely corresponded to the features of NREM parasomnias. First, the presentation was less stereotyped, occurring infrequently and not on a nightly basis. In addition, the affected body regions were localized to the head and neck, with intermittent halting in the progression of movements. Furthermore, these episodes originated during N3 sleep. Those arousals tended to be simple arousal movements (SAMs), which were characterized by three different motor expressions: (a) head flexion/extension, (b) head flexion/extension and limb movement, and (c) head flexion/extension and partial trunk flexion/extension, which are the simplest and briefest DOA patterns (7). Two pieces of research have shown that SWS fragmentation and slow/mixed arousal indexes based on vPSG are two relevant biomarkers for the diagnosis of DoA in adults and children. A 6.8/h cutoff value for the SWS fragmentation index reached a sensitivity and specificity of about 80% in adults, whereas the slow/mixed arousal index had a sensitivity of 94% for the 2.5/h cutoff and 100% specificity for 6/h (9, 10). The CAP rate is a marker of sleep instability (11).

Genetic analysis was conducted on the family, including whole exome sequencing of the DNA coding region. The results revealed a heterozygous mutation (c.2849G>A) in the *KCNT1* gene in the patient (*KCNT1*: NM_020822.3: exon25: c.2849G>A: p.R950Q mutation: pathogenic), and the younger brother carried the same mutation. The mother’s sample showed a low proportion of mutations, and the possibility of her carrying a chimeric mutation could not be ruled out. The father did not possess this mutation (Supplementary Figure S1).

Considering the aforementioned information, the final diagnosis of the patient was *KCNT1*-related SHE combined with DoA. The seizures essentially subsided after 2 months of oxcarbazepine and levetiracetam treatment, although isolated confusional arousals continued to occur intermittently. This further corroborated our diagnosis.

## Discussion

We reported a 17 years-old male patient from a *KCNT1* mutation family who presented with complex abnormal behaviors during sleep. These behaviors were ultimately identified as SHE combined with NREM parasomnias associated with the *KCNT1* mutation. This case underscores the importance for clinicians to thoroughly inquire about the details of paroxysmal events related to sleep disturbances, including their frequency, the presence of tonic abnormalities, the continuity of movements, the sleep stage during which they occur, and so on. Additionally, the absence of epileptiform patterns on EEG could not rule out the diagnosis of SHE.

Since familial events are common in NREM parasomnias, genetic factors are thought to be involved in their occurrence, but the mode of inheritance remains unclear (12). The higher frequency of arousal disorders in families with SHE suggests an intrinsic link between NREM parasomnias and SHE. Approximately 12% of the ADSHE families carry mutations on genes coding for subunits of the heteromeric neuronal nicotinic acetylcholine receptors (nAChRs) (13). Currently, only the ADSHE family carrying mutant nAChRs has been reported to have comorbid NREM parasomnias, suggesting the presence of an abnormal arousal system, possibly with a cholinergic basis, as a shared pathophysiologic mechanism (14). To date, there have been no reports of NREM parasomnias in ADSHE cases with mutant *KCNT1*. Therefore, in this case, we present a novel potential link between NREM parasomnias and *KCNT1* mutations.

The subcellular localization of KNa1.1 in the rat brain indicated a relatively high level of distribution in regions such as the subthalamic nucleus, frontal cortex, reticular formation, cerebellum, and hippocampus (5, 15). This suggests increased excitability within the associated neural networks. The brainstem reticular structure-thalamus-cortex network is closely involved in arousal activities, indicating that *KCNT1*-related disorders may aberrantly activate this network, leading to arousal disturbances. Furthermore, the Slack channels expressed in HEK293 cells and neuronal sodium-activated potassium channels were activated by NAD^+^ and NADP^+^. NAD^+^ levels exhibit circadian oscillations, reflected in EEG as changes in CAPs, which serve as biomarkers of arousal instability during NREM sleep (16). However, it remains to be determined whether the mutant protein in *KCNT1*-related disorders exhibits altered sensitivity to NAD^+^ or NADP^+^. Further studies are needed to confirm these findings.

In conclusion, our study provides a reasoning process to evaluate unusual nocturnal behaviors and demonstrates that *KCNT1*-related SHE may combine with NREM parasomnias, expanding the phenotypic spectrum of *KCNT1* mutations. Additionally, we propose a novel potential association between NREM parasomnias and *KCNT1* mutation.

## Data availability statement

The datasets presented in this article are not readily available because of ethical and privacy restrictions. Requests to access the datasets should be directed to the corresponding author.

## Ethics statement

Ethical review and approval was not required for the study on human participants in accordance with the local legislation and institutional requirements. Written informed consent from the patients/participants or patients/participants’ legal guardian/next of kin was not required to participate in this study in accordance with the national legislation and the institutional requirements. Written informed consent was obtained from the individual(s), and minor(s)’ legal guardian/next of kin, for the publication of any potentially identifiable images or data included in this article.

## Author contributions

DS: Data curation, Formal analysis, Writing – original draft. YL: Formal analysis, Writing – original draft, Validation. XL: Formal analysis, Writing – original draft, Data curation. JL: Formal analysis, Methodology, Software, Writing – review & editing. WL: Writing – review & editing, Conceptualization.
