# Supplementary figures and images for: Case report: A young man with non-rapid eye movement parasomnias in a KCNT1-related epilepsy family
Source: Front Neurol. 2023 Nov 24;14:1280348. doi: 10.3389/fneur.2023.1280348 (PMC10704451; doi:10.3389/fneur.2023.1280348)

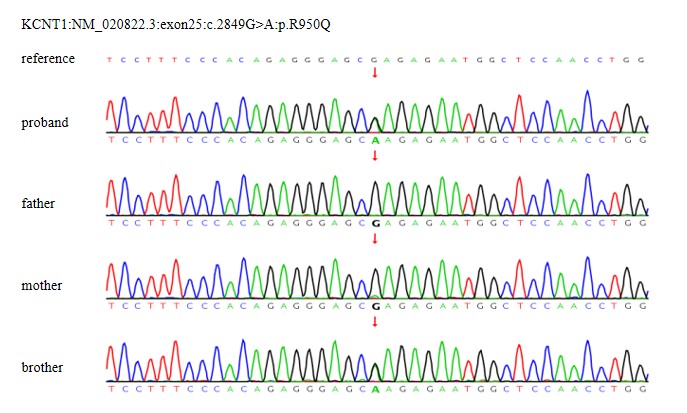

Supplement: Supplementary file 4 [file Image_1.JPEG]
